# Is the Doctor a Shylock?

**Published:** 1907-07

**Authors:** 


### IS THE DOCTOR A SHYLOCK?

### PHYSICIANS INCOMES.

Edgar Allen Forbes has investigated this subject and claims that a very few physicians of pre-eminence have large incomes, but smaller than men pre-eminent in other lines.

Something like 20 per cent. of the physicians in the larger cities have handsome incomes, in return for expert work and much of it.

Eighty per cent. of city physicians and most of the country doctors make little more than a decent living—many not even that.

The average of fees charged in city and in country is no larger than it was twenty-five years ago.

The code of medical ethics (established by physicians themselves) is such that no class of the American people need suffer for medical attention because of inability to pay.

Is the doctor a Shylock?—The World's Work, May, 1907.

